# System level identification and multidimensional analysis of hub genes reveal complex regulatory networks underlying human iPSC differentiation into motor neurons

**DOI:** 10.1016/j.bbrep.2026.102738

**Published:** 2026-08-12

**Authors:** Maryam Sadeghi, Shima Hadifar, Abozar Ghorbani

**Affiliations:** a Department of Biology-Genetics, Science and Research Branch, Azad University of Tehran, Iran; b Department of Bacteriology, Pasteur Institute of Iran, Tehran, Iran; c Nuclear Agriculture Research School, Nuclear Science and Technology Research Institute (NSTRI), Karaj, Iran

**Keywords:** iPSCs, Motor neuron differentiation, Hub genes, miRNA-mRNA interactions, Codon usage bias, Systems biology

## Abstract

Human-induced pluripotent stem cells (iPSCs) hold considerable potential for generating motor neurons (MNs), offering new avenues for disease modeling and regenerative medicine. To elucidate the molecular mechanisms underlying iPSC differentiation into MNs, we conducted an integrated bioinformatics analysis of transcriptomic data from the mature Day 28 stage of a publicly available 28-day iPSC differentiation dataset**.** Protein-protein interaction (PPI) networks were constructed using STRING and visualized in Cytoscape, while CytoHubba identified 15 hub genes, including BUB1, TOP2A, AURKB, CCNA2, and TP53, which are primarily associated with cell cycle regulation, mitotic progression, and genomic stability. Gene Ontology (GO) enrichment analysis identified biological functions related to chromosomal organization, cytoskeletal remodeling, metabolic activity, and translational regulation, whereas Kyoto Encyclopedia of Genes and Genomes (KEGG) pathway analysis highlighted cell cycle, DNA replication, cellular senescence, p53 signaling, and oocyte meiosis. CytoCluster analysis identified distinct subnetwork modules linked to neuronal differentiation. Promoter motif analysis via MEME uncovered conserved transcription factor binding sites, whereas miRNA-mRNA interaction prediction using the psRNATarget database identified 5316 potential regulatory pairs that may fine-tune gene expression during MN maturation. Codon usage analysis of 163 genes indicated moderate to high translational optimization, likely influenced by both mutational bias and selective codon preferences, with GC3 content strongly affecting codon choice. These patterns suggest potential mechanisms that may promote efficient protein synthesis during MN differentiation; however, experimental validation is required. This systems-level bioinformatics framework integrates multiple computational analyses to characterize transcriptional, post-transcriptional, and translational regulatory mechanisms associated with human MN differentiation. However, as these results are derived from bioinformatics analyses, they warrant further experimental validation and have the potential to advance our understanding of the molecular interactions and signaling pathways that regulate MN differentiation and maturation. These findings provide a computational framework for exploring regulatory mechanisms of motor neuron differentiation and prioritizing candidate genes for future experimental validation.

## Highlights

• Systems analysis of iPSC motor neuron differentiation.
• Identification of 15 key hub regulatory genes.
• Enrichment of cell cycle and p53 signaling pathways.
• Integrated miRNA–mRNA regulatory network prediction.
• Codon usage bias suggests translational optimization.

## 1 Introduction

Human induced pluripotent stem cells (iPSCs) possess remarkable potential for differentiation into diverse neural lineages, offering valuable prospects for disease modeling and regenerative medicine. However, their clinical application remains constrained by risks such as tumorigenesis and unpredictable genetic or epigenetic alterations. Notably, the advent of iPSC technology has enabled the generation of iPSC-derived motor neurons (MNs), providing powerful platforms for studying neurodegenerative disorders and developing cell-based therapies [1,2]. Neural commitment and subsequent differentiation are complex processes governed by dynamic gene regulatory networks.

Despite extensive transcriptomic diversity in neural tissues, comprehensive analyses of gene expression across differentiation stages remain limited. Such studies are crucial for understanding molecular mechanisms that regulate neuronal specification and maturation, and for developing therapeutic strategies against neurodegenerative diseases such as Parkinson's and Alzheimer's [3,4]. Single-cell RNA sequencing analyses have further revealed that in vitro differentiation of iPSCs often yields heterogeneous MN populations, emphasizing the need for improved differentiation protocols [5,6].

Various in vitro methods have been established to induce iPSC differentiation into MNs; however, prolonged maturation and heterogeneity continue to pose major challenges [7]. Two-dimensional culture models are useful for generating relatively homogeneous MNs and investigating cell-autonomous mechanisms, whereas more complex systems are required to study non-cell-autonomous processes, including motor circuit formation and prion-like propagation [8]. Comparative analyses of patient-derived and control iPSC-MNs have elucidated molecular mechanisms underlying MN diseases and facilitated high-throughput drug screening. Nevertheless, variability among iPSC lines arising from both genetic and non-genetic factors remains a critical limitation. Studies have shown that up to 50% of expression variability in iPSCs is attributable to genetic background, highlighting the importance of standardized reprogramming and differentiation protocols [9,10].

Genome-wide transcriptomic profiling of iPSC differentiation into MNs provides critical insights into the molecular cascades that orchestrate neuronal lineage commitment and maturation [9]. The differentiation process is governed by intricate protein-protein interaction (PPI) networks, where regulatory complexes coordinate transcriptional and post-transcriptional programs essential for neuronal identity and axonal functionality. Functional annotation through Gene Ontology (GO) enrichment enables the systematic characterization of biological processes, molecular functions, and cellular components associated with genes driving MN specification [11].

MicroRNAs (miRNAs) represent an additional regulatory layer that refines gene expression dynamics during motor neuron differentiation. Through sequence-specific binding to the 3′ untranslated regions (3′UTRs) of target mRNAs, miRNAs modulate transcript stability and translational output, thereby influencing neuronal morphogenesis and synaptic maturation. iPSC-derived MNs models provide a powerful experimental system to dissect these miRNA-mediated regulatory mechanisms within a human context. Beyond transcriptional and post-transcriptional regulation, codon usage bias represents an additional layer of control by impacting mRNA stability, translation efficiency, and protein folding. Analysis of codon usage patterns, therefore, offers perspectives on the evolutionary and functional optimization of gene expression during iPSCs to MNs conversion [[12], [13], [14]].

In this study, we employed an integrated, multi-layered bioinformatic framework to analyze transcriptomic data from a Day 28 iPSCs-to-MNs differentiation model. By analyzing differentially expressed genes from the mature motor neuron stage and constructing interaction networks, we identified candidate hub genes and regulatory modules involved in MNs lineage progression. Functional enrichment, promoter motif discovery, miRNA target prediction, and codon usage analysis collectively provide a comprehensive view of the transcriptional, post-transcriptional, and translational mechanisms governing human MNs differentiation. These findings establish a computational framework for future experimental validation and the development of therapeutic strategies for neurodegenerative and neuromuscular disorders.

## 2 Materials and methods

### 2.1 RNA-seq data retrieval and computational analysis

Data on genes involved in Human-induced pluripotent stem cells (iPSCs) differentiation to motor neurons (MNs) were retrieved from exclusively computational analysis of transcriptomic data publicly available in NCBI, published by Solomon et al. (2021) [9]. In this article, the dataset encompassed five key developmental stages: iPSCs (Day 0), neural stem cells (Day 7), motor neuron progenitors (Day 13), early motor neurons (Day 18), and mature motor neurons (Day 28). Day 28 was selected as representing the mature motor neuron stage, enabling identification of terminal differentiation regulatory networks. Differential expression results were obtained from the supplementary dataset reported bySolomon et al. (2021) [9]. Differentially expressed genes (DEGs) on Day 28 were considered significant with |log2FC| > 2 and *P*-value < 0.001.

### 2.2 Construction of protein-protein interaction (PPI) networks and hub gene analysis

PPI analysis was performed using the STRING database (version 12; http://string-db.org) with *Homo sapiens* selected as the reference organism. Interactions with a combined confidence score >0.700 (high confidence) were retained for network construction in order to explore potential interactions among the selected genes [15]**.** STRING enrichment analyses were considered statistically significant at p < 0.05. The resulting interaction network was subsequently imported into Cytoscape (version 3.10.4) for topological refinement and module detection [16]. Hub genes within the network were identified using the CytoHubba plugin (version 0.1), which ranks nodes based on their topological significance in biological networks. Four node-ranking algorithms, Degree (Deg), Maximal Clique Centrality (MCC), Maximum Neighborhood Component (MNC), and Density of Maximum Neighborhood Component (DMNC), were applied to evaluate node importance from complementary topological perspectives. Genes consistently ranked among the top candidates across each of the four algorithms were considered hub genes and selected for subsequent analyses. [17]. These hub genes were subsequently analyzed in STRING to predict subnetworks, which were visualized again in Cytoscape to facilitate interpretation of core protein interactions [18,19].

### 2.3 Gene Ontology and pathway enrichment analysis in the subnetwork

In this study, comprehensive functional enrichment analysis was carried out using the STRING web-based platform, incorporating six major annotation categories: Biological Processes (BP), Cellular Components (CC), Molecular Functions (MF), Tissue Expression (TISSUES), Disease-Gene Associations (DISEASE), and Kyoto Encyclopedia of Genes and Genomes (KEGG) Pathways. These integrative analyses enabled a systematic exploration of gene function, providing insights into the biological roles, cellular localization, and molecular mechanisms of the differentially expressed genes. The tissue expression enrichment analysis offered valuable information on the predominant tissues and organ systems in which these genes are active, while the disease-gene association analysis highlighted potential links between hub genes and known pathological conditions. Collectively, these results contributed to a more comprehensive understanding of the functional relevance and biological context of the hub genes. Furthermore, KEGG pathway analysis was employed to elucidate the major signaling and metabolic pathways associated with the identified subnetworks [20,21].

### 2.4 Cluster analysis of the network

Network clustering was performed using the Cytocluster plugin (version 2.1.0) in Cytoscape to identify densely connected regions within the PPI network [22]. The Protein Complex Identification Algorithm (IPCA) was applied to further analyze subnetworks and detect potential protein complexes [23]. IPCA evaluates the weight of each edge based on the number of shared neighbors between connected nodes and assigns node weights using a threshold value of 10. This threshold serves as a tuning parameter to filter nodes according to their connectivity strength, thereby enhancing the biological relevance and precision of protein complex detection. Subsequently, genes within each identified cluster were subjected to KEGG pathway enrichment analysis using the STRING database to elucidate the major signaling and metabolic pathways associated with each module.

### 2.5 Cis-regulatory motif detection and functional annotation

To explore transcriptional regulatory factors of hub genes at the promoter level, one-kilobase upstream promoter regions of hub genes were retrieved from Ensembl (https://www.ensembl.org) and analyzed using the MEME Suite (version 5.5.9) (https://meme-suite.org/meme/tools/gomo). Statistically significant conserved sequence motifs were discovered under stringent E-value and P-value thresholds (P < 0.01 and E < 0.01), and motif redundancy was eliminated through cross-comparison with known transcription factor binding motifs s using the Tomtom algorithm against the JASPAR CORE database (release 2026). Functional annotation of the identified motifs was subsequently inferred using the GOMo (Gene Ontology for motifs) tool implemented in MEME Suite (version 5.5.9), revealing possible regulatory functions involved in neuronal identity establishment and neuronal maturation [[24], [25], [26]].

### 2.6 Identification of potential miRNAs targeting hub gene

Potential post-transcriptional regulatory interactions were examined by querying hub gene sequences against human miRNAs retrieved from the miRBase repository (https://www.mirbase.org) using the psRNATarget platform (https://www.zhaolab.org). Predictions were generated under default sensitivity parameters with a maximum expectation value of 4, and the resulting candidate miRNAs were cross-referenced with previously reported human miRNAs to enhance biological relevance [26].

### 2.7 Codon usage bias analysis

To further characterize the molecular behavior of hub genes at the translational level, codon usage bias was analyzed by extracting full open reading frames (ORF) and quantifying codon usage indices including codon adaption index (CAI), effective number of codons (ENC), GC composition, GC content at the third synonymous codon position (GC3S), and Relative Synonymous Codon Usage (RSCU) using custom R scripts (R version 4.3.1). CAI was used as an indicator of the extent to which preferred codons are utilized and has been associated with potential translational efficiency. ENC was employed to estimate the degree of codon usage bias, ranging from 20 (extreme bias) to 61 (no bias). GC and GC3S were used to assess nucleotide compositional constraints, whereas RSCU was calculated to identify preferential usage of synonymous codons independent of amino acid composition. These analyses made it possible to explore the relationship between expression level, codon preference, and potential translational efficiency during the course of iPSCs to MNs differentiation [27,28].

## 3 Results

### 3.1 Identification of differentially expressed genes (DEGs)

**Fig. 1:**
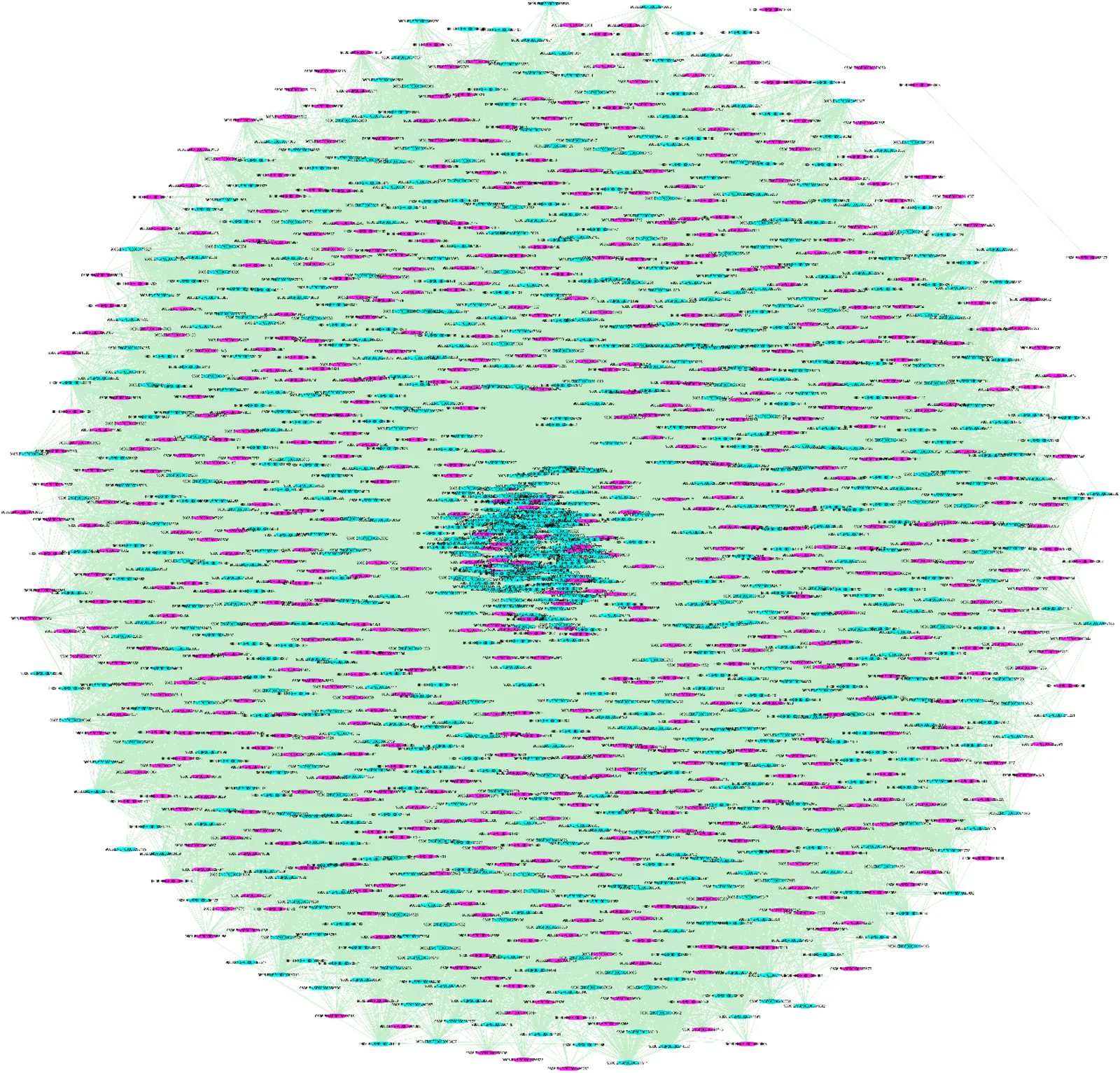
The main Network involved in Human-induced pluripotent stem cell differentiation to motor neurons, identified by STRING and Cytoscape from Day 28.

In the present study, transcriptomic profiling data from Day 28 were analyzed. A total of 3680 genes met the DEG criteria (|log2FC| > 2, P < 0.001) at Day 28, forming the input set for protein–protein interaction network construction. The global interaction network generated through STRING and visualized in Cytoscape displayed a dense and interconnected structure (Fig. 1) [29].

### 3.2 Reconstruction of gene and PPI networks followed by hub gene analysis

**Fig. 2:**
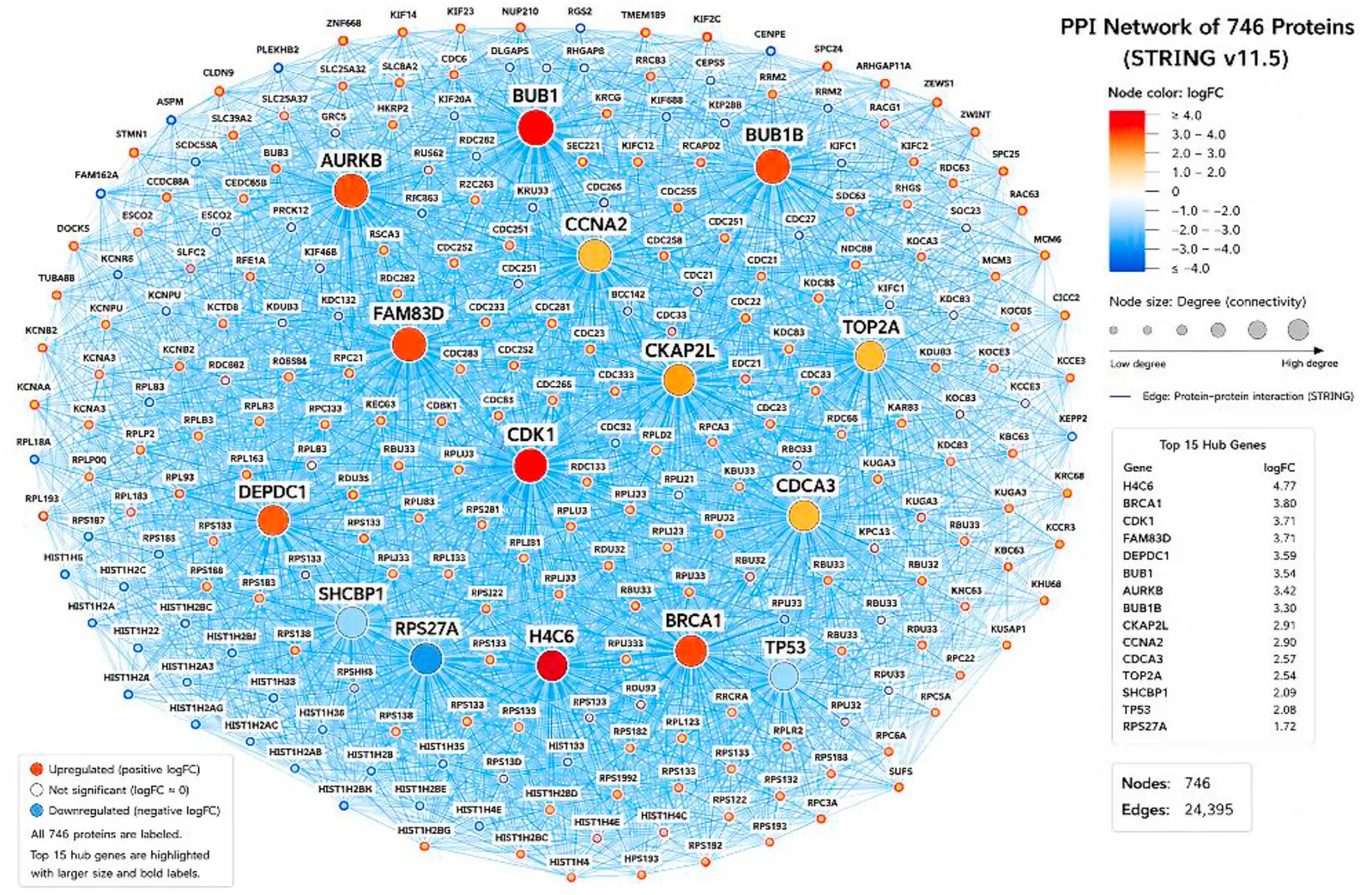
The subnetwork of hub genes involved in Human-induced pluripotent stem cell differentiation to motor neurons on Day 28. Green node colors indicate hub genes selected by cytoHubba.

This study computationally examined regulatory gene interactions associated with the differentiation of human-induced pluripotent stem cells into motor neurons at Day 28 using differentially expressed genes. To characterize the interaction landscape, a PPI network was generated using the STRING database, yielding a network of 746 nodes and 24,395 edges (Fig. 2). The network was subsequently imported into Cytoscape for structural visualization and topological assessment. Hub gene prioritization was conducted using the CytoHubba plugin, applying multiple graph-theoretic ranking approaches. This analysis highlighted 15 genes with high network centrality scores (Table 1). A derived subnetwork of these hub genes comprised 3680 genes, with full annotations provided in (Supplementary File 1). Among the prioritized genes, BUB1, BUB1B, TOP2A, AURKB, and CCNA2 consistently ranked highest under the MCC metric, indicating dense connectivity within the network core. In parallel, CKAP2L emerged as a top candidate under the DMNC algorithm, while TP53, CDK1, RPS27A, BRCA1, and H4C6 were highlighted by MNC- and degree-based scoring strategies (Table 1). All identified hub genes exhibited positive differential expression, with log2 fold-change values ranging from 1.72 to 4.77. From a network-based perspective, the dominance of cell-cycle and genome integrity–related genes suggests a structurally coordinated regulatory module within the inferred differentiation network. These patterns reflect connectivity-driven prioritization rather than experimentally verified regulatory hierarchy, positioning these genes as computational candidates for further investigation.

**Table 1:** Ranking of hub genes identified during the differentiation of Human-induced pluripotent stem cells (iPSCs) into motor neurons (MNs) on Day 28 using the CytoHubba plugin in Cytoscape and STRING.

Gene ID	Hub Algorithm	Rank	Log2 Fold change	Gene Description
BUB1	MCC	1	3.54	Mitotic checkpoint serine/threonine-protein kinase BUB1
BUB1B	MCC	1	3.30	Mitotic checkpoint serine/threonine-protein kinase BUB1 beta
TOP2A	MCC	1	2.54	DNA topoisomerase 2-alpha
AURKB	MCC	1	3.42	Aurora kinase B
CCNA2	MCC	1	2.90	Cyclin-A2
CKAP2L	DMNC	1	2.91	Cytoskeleton-associated protein 2-like
TP53	MNC, Degree	1,1	2.08	Cellular tumor antigen p53
FAM83D	DMNC	2	3.71	Protein FAM83D
CDK1	MNC, Degree	2,2	3.71	Cyclin-dependent kinase 1
SHCBP1	DMNC	3	2.09	SHC SH2 domain-binding protein 1
BRCA1	MNC, Degree	3,3	3.80	Breast cancer type 1 susceptibility protein
CDCA3	DMNC	4	2.57	Cell division cycle-associated protein 3
RPS27A	MNC, Degree	4,4	1.72	Ubiquitin-40 S ribosomal protein S27a
DEPDC1	DMNC	5	3.59	DEP domain-containing protein 1 A;
H4C6	MNC, Degree	5,5	4.77	Histone H4; Core component of nucleosome

### 3.3 Functional pathway and Gene Ontology (GO) enrichment analysis

GO enrichment analysis was applied to functionally annotate genes within the hub gene–associated subnetwork across biological process (BP), molecular function (MF), cellular component (CC), Tissue Expression (TISSUES), Disease-Gene Associations (DISEASE), and KEGG pathways [30,31]. The resulting dataset was evaluated using integrated enrichment frameworks, with complete outputs provided in (Supplementary File 2).

In the molecular function category, the gene set demonstrated pronounced enrichment in ATP binding, microtubule binding, and DNA-dependent ATPase activity (Fig. 4B) [32,33]. Additional significant terms included chromatin binding, tubulin binding, DNA helicase activity, ATP-dependent helicase activity, and microtubule motor activity, all with extremely strong statistical support (FDR < 1 × 10^−11^). This enrichment profile points to a concentration of energy-dependent enzymatic activities coupled with cytoskeletal dynamics within the analyzed gene set [34,35].

Within the biological process category, strong enrichment was observed in fundamental cellular and metabolic programs (Fig. 4A), including organic substance metabolism, cellular metabolism, nitrogen compound metabolism, and macromolecule biosynthesis, all reaching extremely low FDR values (<1.0 × 10^−90^). The largest gene representation was detected in cellular metabolic and macromolecule biosynthetic processes (>100 genes), indicating broad engagement in essential biosynthetic and regulatory cellular functions [36].

Cellular component analysis revealed significant enrichment across intracellular structures, including cytoplasm, nucleus, membrane-bound organelles, and protein-containing complexes (FDR < 1.0 × 10^−40^). The most prominent gene clusters localized to intracellular organelles and cytoplasmic regions (Fig. 4C), suggesting distributed functional activity across multiple subcellular compartments.

**Fig. 3:**
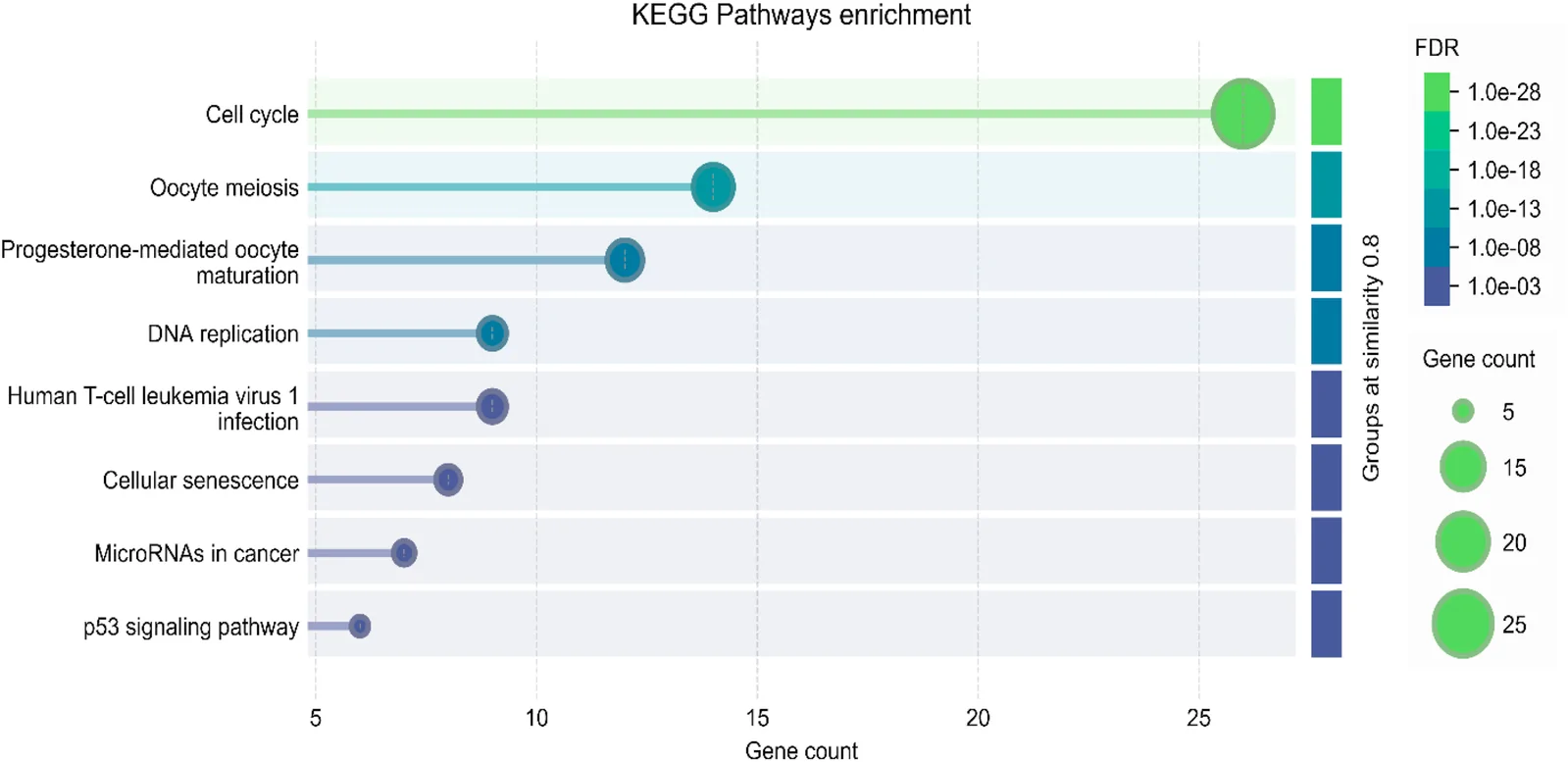
Kyoto Encyclopedia of Genes and Genomes (KEGG) pathway enrichment analysis of hub genes associated with subnetwork genes. The analysis was performed using STRING (version 12) to identify significantly enriched biological pathways according to the KEGG database.

KEGG pathway enrichment analysis identified the cell cycle as the most significantly enriched pathway (FDR = 2.51 × 10^−28^), followed by oocyte meiosis, progesterone-mediated oocyte maturation, DNA replication, cellular senescence, p53 signaling, Human T-cell leukemia virus 1 infection, and microRNAs in cancer (Fig. 3). These enriched pathways are predominantly associated with cell-cycle regulation, mitotic progression, chromosome segregation, DNA replication, and checkpoint control, consistent with the known biological functions of the identified hub genes [32,33,[37], [38], [39]]. Collectively, these findings suggest that regulatory mechanisms governing cell proliferation and genomic stability constitute prominent features of the inferred molecular network associated with motor neuron differentiation [40,41].

**Fig. 4:**
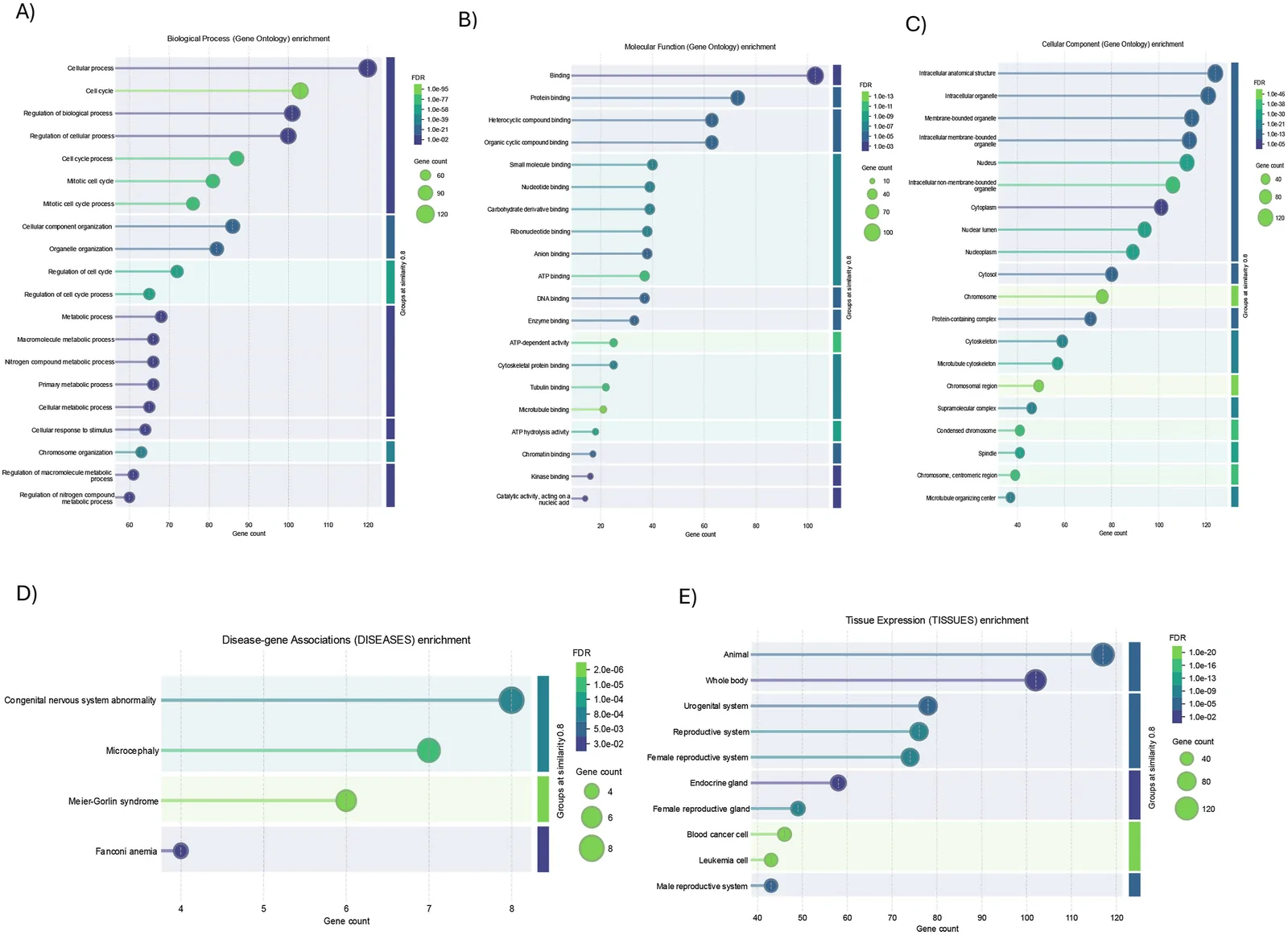
Integrated Gene Ontology (GO) and functional enrichment analysis of hub gene subnetworks derived from the protein–protein interaction (PPI) network. The analysis, performed using STRING (version 12), includes GO enrichment of Biological Process (BP), Molecular Function (MF), and Cellular Component (CC), as well as tissue expression and disease–gene association enrichment, highlighting the functional roles, subcellular localization, and disease relevance of the identified hub genes.

Tissue enrichment analysis showed strong associations with liver, testis, and brain tissues (Fig. 4D), each displaying highly significant enrichment signals (FDR ≤ 1 × 10^−20^) [30,31]. Disease association analysis further linked the gene set to cancer-related conditions, neurodegenerative disorders, and metabolic diseases (FDR ≤ 2 × 10^−6^) [42], reflecting shared annotation structure across public gene–phenotype databases (Fig. 4E).

Overall, the enrichment landscape suggests a coordinated involvement of metabolic, translational, and structural gene programs within the inferred network, providing a systems-level computational view of potential regulatory organization during motor neuron differentiation.

### 3.4 Cluster analysis of the PPI network

Cluster analysis of the protein–protein interaction (PPI) network was conducted to delineate functional modules and highly interconnected regions associated with genes implicated in iPSC differentiation into motor neurons. The CytoCluster plugin, which incorporates six clustering strategies, was applied, and the IPCA algorithm was selected to detect densely interconnected subnetworks within the global interaction map. Subnetwork decomposition using STRING revealed three major clusters ranked by size and interaction density (Table 2). Cluster 1 contained 150 nodes and 7188 edges, Cluster 2 included 147 nodes and 7207 edges, and Cluster 3 consisted of 144 nodes and 6600 edges, collectively reflecting a highly compact and modular network architecture. Functional annotation of these clusters through KEGG pathway mapping showed that all three modules converged on core regulatory processes, including cell cycle control, DNA replication, oocyte meiosis, cellular senescence, the p53 signaling pathway, and microRNAs in cancer, indicating shared involvement in fundamental proliferative and genomic maintenance programs. Cluster 1 displayed broader pathway diversity, with additional representation of FoxO, PI3K-Akt, HIF-1, and ErbB signaling cascades, as well as apoptosis-related pathways. Moreover, this cluster showed strong links to multiple cancer-associated pathways, including breast, colorectal, pancreatic, gastric, and hepatocellular carcinoma, alongside infection-related pathways such as Epstein–Barr virus, HIV-1 infection, and Kaposi sarcoma-associated herpesvirus. In contrast, Clusters 2 and 3 were more functionally constrained, primarily mapping to canonical cell cycle regulation and differentiation-associated pathways, with comparatively limited association with oncogenic or viral infection signaling. This suggests a gradation in functional specialization across clusters, with Cluster 1 representing a more pathway-diverse regulatory module.

**Table 2:** Summary of clusters (rank 1 to 3) resulting from cluster analysis of the subnetwork of hub genes in iPSC differentiation to MNs using the CytoCluster app and STRING.

Cluster	Nodes	Edges	KEGG for each pathway	
1	150	7188	Cell cycle	p53 signaling pathway
			MicroRNAs in cancer	Viral carcinogenesis
			Oocyte meiosis	Endocrine resistance
			Cellular senescence	Prostate cancer
			Human T-cell leukemia virus 1 infection	Pathways in cancer
			Progesterone-mediated oocyte maturation	DNA replication
			FoxO signaling pathway	Breast cancer
			Human papillomavirus infection	Bladder cancer
			Transcriptional misregulation in cancer	Platinum drug resistance
			Small- cell lung cancer	Pancreatic cancer
			Non-small cell lung cancer	Gastric cancer
			Epstein-Barr virus infection	Kaposi sarcoma-associated herpesvirus infection.
			Glioma	Endometrial cancer
			Melanoma	HIF-1 signaling pathway
			Human immunodeficiency virus 1 infection	Thyroid cancer
			Thyroid hormone signaling pathway	Hepatocellular carcinoma
			Colorectal cancer	Chronic myeloid leukemia
			Shigellosis	ErbB signaling pathway
			Adherens junction	Apoptosis
			EGFR tyrosine kinase inhibitor resistance	Fanconi anemia pathway
			Ubiquitin- mediated proteolysis	Proteoglycans in cancer
			PI3K-Akt signaling pathway	Mitophagy - animal
			Alcoholism	Renal cell carcinoma
			AGE-RAGE signaling pathway in diabetic complications	Acute myeloid leukemia
			Cushing syndrome	Central carbon metabolism in cancer
			Human cytomegalovirus infection	
2	147	7207	term description	
			Cell cycle	
			Oocyte meiosis	
			Progesterone-mediated oocyte maturation	
			DNA replication	
			Cellular senescence	
			Human T-cell leukemia virus 1 infection	
			p53 signaling pathway	
			MicroRNAs in cancer	
3	144	6600	term description	
			Cell cycle	
			Oocyte meiosis	
			Progesterone-mediated oocyte maturation	
			DNA replication	
			Cellular senescence	
			Human T-cell leukemia virus 1 infection	
			p53 signaling pathway	
			MicroRNAs in cancer	

### 3.5 Identification of potential miRNA regulators of hub genes

**Fig. 5:**
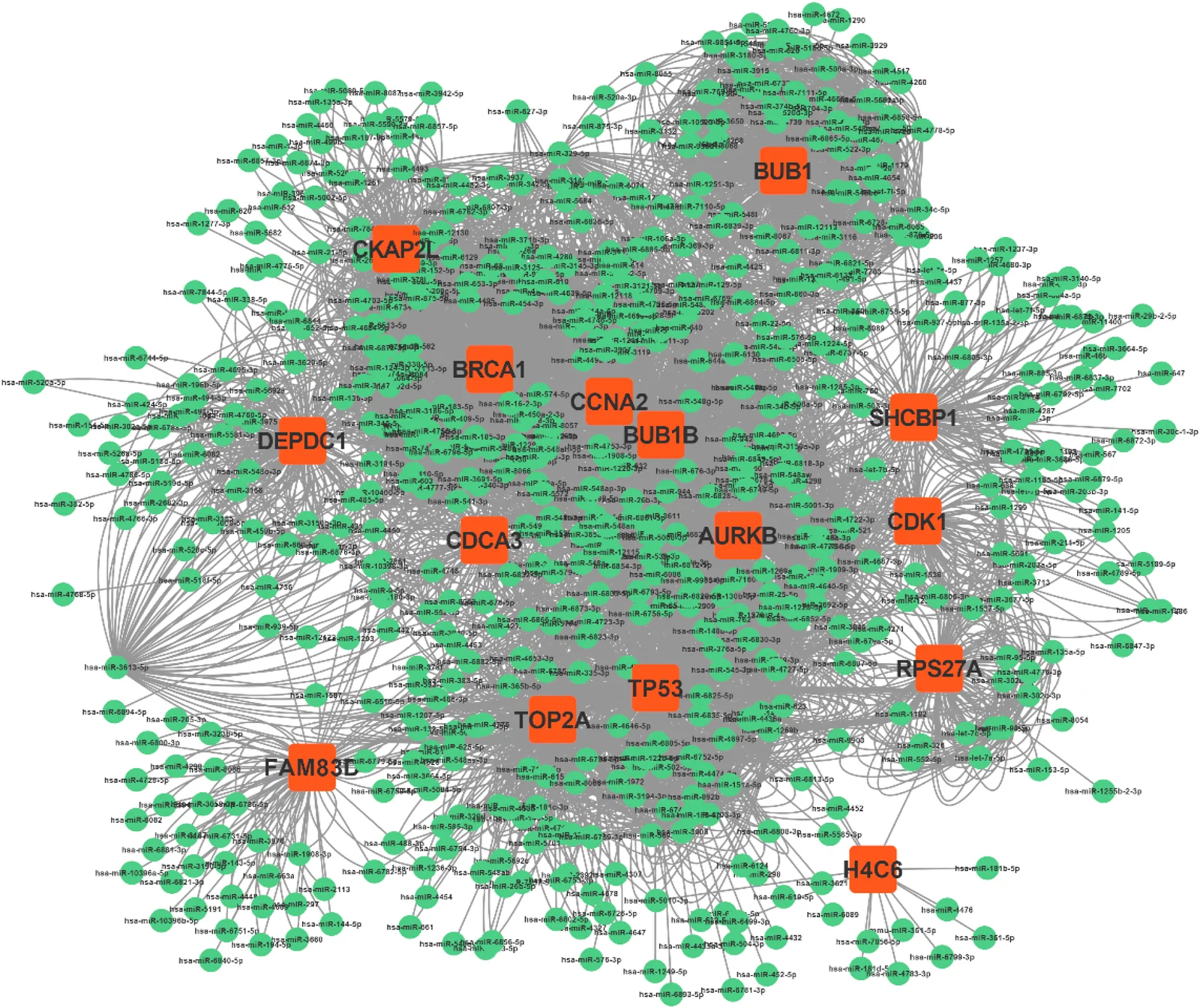
Potential miRNAs targeting hub genes involved in iPSC differentiation to MNs were predicted using psRNATarget.

PsRNATarget analysis was conducted to predict miRNA–hub gene regulatory interactions potentially involved in iPSC differentiation into motor neurons. The results indicated that several hub genes related to cell-cycle control and genomic stability, including BRCA1, TP53, BUB1B, BUB1, and TOP2A, were predicted to be targeted by multiple distinct miRNAs (Fig. 5), highlighting their central position within the inferred post-transcriptional regulatory landscape. Among the predicted regulators, a subset of miRNAs exhibited broad targeting breadth across the hub gene set. In particular, hsa-miR-3613-5p showed predicted interactions with eight hub genes, including BRCA1, BUB1B, and TOP2A, while hsa-miR-329-5p was linked to six genes primarily involved in DNA repair and cell-cycle progression [43]. Additional miRNAs, such as hsa-miR-650 and hsa-miR-762**,** were predicted to regulate genes implicated in both mitotic control and cytoskeletal organization, suggesting coordinated post-transcriptional modulation of proliferative and structural programs. Functional annotation of miRNA-targeted hub genes further indicated strong representation of processes related to cell-cycle phase transition, chromosome segregation, and mitotic nuclear division [43]. In addition, genes involved in spindle organization and microtubule dynamics, including AURKB, FAM83D, and CKAP2L, were also recovered within the predicted miRNA interaction set, reinforcing their integration into mitotic regulatory networks. Network topology assessment showed that BRCA1 and TP53 occupied highly connected positions within the miRNA–mRNA interaction network, reflecting extensive predicted regulatory input from multiple miRNAs [43,44].

### 3.6 Promoter motif analysis of hub genes

Upstream flanking regions (URFs), defined as 1 kbp sequences upstream of each hub gene, were analyzed to characterize conserved sequence motifs and putative cis-regulatory elements (CREs) potentially involved in transcriptional regulation. Promoter sequences were retrieved from the Ensembl database and subjected to de novo motif discovery using MEME software. This analysis identified six statistically significant conserved motifs ranging in length from 14 to 674 bp (Table 3) [45]. The identified motifs were further compared with known transcription factor binding motifs using Tomtom, and functional associations were explored using GOMo. GOMo-based annotation suggested that several motifs were associated with terms related to transcriptional regulation, chromosome organization, G-protein-coupled receptor signaling, defense response, and Wnt receptor signaling pathways [24]. Among the hub genes, CDK1, AURKB, and FAM83D showed distinct motif association profiles. CDK1 was associated with three predicted motifs, whereas AURKB showed broader motif representation across multiple predicted motifs. FAM83D was associated with motif MA2398.1, which was linked to transcriptional activation and Wnt receptor signaling-related annotations [46]. Overall, these findings suggest potential differences in promoter-level regulatory features among selected hub genes within the inferred transcriptional network.

**Table 3:** The conserved motifs found in promoters of the hub gene by MEME analysis.

Motif	Logo	Predictions	Top 5 specific predictions
MA0277.1		15	MF olfactory receptor activity. BP sensory perception of smell. BP G-protein coupled receptor protein signaling pathway. BP regulation of chromosome organization BP response to stimulus.
MA0537.1		15	MF olfactory receptor activity BP sensory perception of smell BP G-protein coupled receptor protein signaling pathway BP regulation of chromosome organization BP response to stimulus
MA1267.2		14	MF olfactory receptor activity BP sensory perception of smell BP G-protein coupled receptor protein signaling pathway BP defense response MF taste receptor activity
MA1268.2		15	MF olfactory receptor activity BP sensory perception of smell BP G-protein coupled receptor protein signaling pathway BP regulation of chromosome organization BP response to stimulus
MA1274.1		15	MF olfactory receptor activity BP sensory perception of smell BP G-protein coupled receptor protein signaling pathway BP regulation of chromosome organization BP response to stimulus
MA1278.2		14	MF olfactory receptor activity BP sensory perception of smell BP G-protein coupled receptor protein signaling pathway BP defense response MF taste receptor activity
MA1279.2		15	MF olfactory receptor activity BP sensory perception of smell BP G-protein coupled receptor protein signaling pathway BP defense response MF taste receptor activity
MA1281.2		14	MF olfactory receptor activity BP sensory perception of smell BP G-protein coupled receptor protein signaling pathway BP defense response MF taste receptor activity
MA2398.1		674	BP negative regulation of signal transduction CC cytosol MF transcription activator activity CC transcription factor complex BP Wnt receptor signaling pathway

### 3.7 Codon usage bias and translational efficiency analysis

Codon preference patterns among the identified hub genes were evaluated using previously published sequencing data through multiple computational metrics. Codon usage bias was assessed using the codon adaptation index (CAI), effective number of codons (ENC), GC content, and GC content at the third codon position (GC3S). Relationships among these indices were further examined to identify the primary determinants of codon usage variation (Supplementary File 3).

**Fig. 6:**
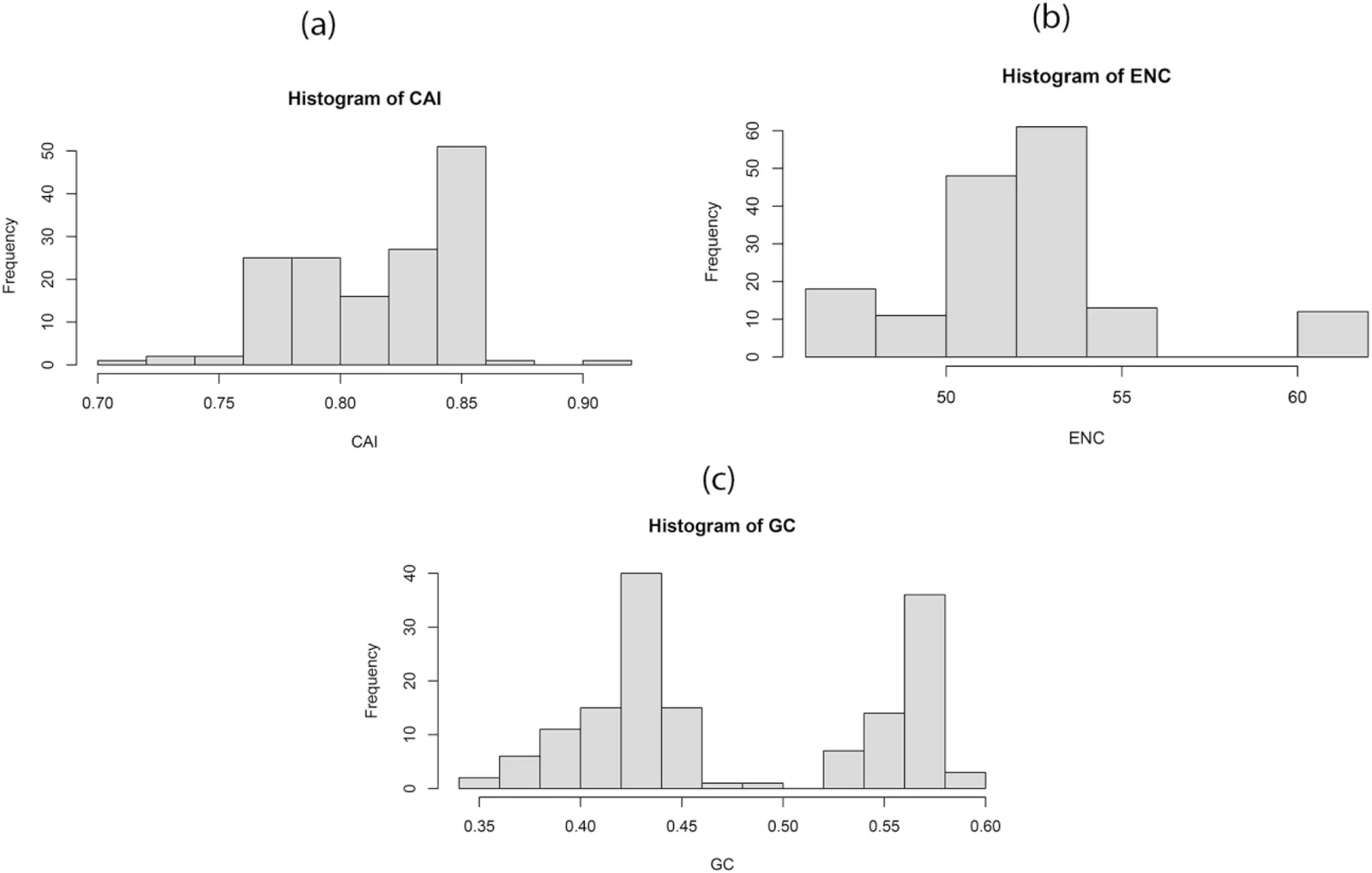
Distribution of GC content, codon adaptation index (CAI), and effective number of codons (ENC) among 163 analyzed genes, indicating moderate codon usage bias and variable levels of codon optimization. Analyses were performed using custom R scripts.

A total of 163 gene sequences were analyzed using Pearson's correlation to quantify associations among codon usage parameters (Supplementary Table 3). CAI values ranged from 0.72 to 0.91 (mean = 0.815), indicating generally moderate to high levels of translational adaptation (Fig. 6a). ENC values varied between 46.2 and 61.0 (mean = 52.19), reflecting an intermediate degree of codon usage bias (Fig. 6b). GC content ranged from 35.6% to 59.9% (mean = 47.8%), while GC3S values spanned 26.3% to 72.9% (mean = 48.0%) [47], suggesting notable variability in nucleotide composition across coding regions.

Correlation analysis revealed structured relationships among codon usage features. CAI showed strong inverse associations with GC content (r = −0.82) and GC3S (r = −0.75), indicating that higher translational adaptation scores were generally associated with lower GC enrichment. ENC exhibited moderate negative correlations with GC (r = −0.44) and GC3S (r = −0.60), suggesting that nucleotide composition partially influences codon bias strength. In contrast, a strong positive correlation was observed between GC and GC3S (r = 0.92), highlighting a close compositional dependency between overall genomic GC content and third-position nucleotide usage (Fig. 6c) [48].

Relative synonymous codon usage (RSCU) analysis further indicated non-random codon selection across the examined gene set (Supplementary File 4). Within the phenylalanine codon family, TTT (RSCU = 1.28) was preferentially used over TTC (RSCU = 0.72). A similar pattern was observed in leucine codons, where TTG (RSCU = 1.08) was slightly favored compared to TTA (RSCU = 0.92). A more pronounced bias was observed for serine, with TCT showing the highest relative usage within its codon family (RSCU = 1.88). Notably, codons with higher weighted CAI values tended to correspond with elevated RSCU scores, indicating concordance between translational efficiency metrics and synonymous codon preference patterns [49,50].

## 4 Discussion

The identification of 15 hub genes in the mature motor neuron stage (Day 28) suggests that cell-cycle regulation, mitotic control, and genomic stability are central features of the inferred regulatory network underlying iPSC differentiation into motor neurons. The consistent emergence of genes such as BUB1, BUB1B, TOP2A, AURKB, CCNA2, CKAP2L, and TP53 across multiple centrality measures indicates that these nodes may represent structurally important elements within the protein–protein interaction network rather than isolated expression changes. However, because the hub gene analysis was based primarily on the mature Day 28 stage and was not derived from temporal network modeling across all differentiation stages, these genes should be interpreted as candidate regulators associated with the Day 28 differentiation state rather than confirmed dynamic regulators of the entire iPSC-to-motor neuron differentiation trajectory.

Several of the identified hub genes are well-known regulators of mitotic progression and DNA integrity. BUB1 and BUB1B encode spindle assembly checkpoint kinases that ensure proper chromosome segregation [33,34], suggesting that checkpoint-related signatures may remain detectable in the Day 28 network. TOP2A, a DNA topoisomerase [35], and AURKB, a key mitotic kinase involved in chromosome alignment and cytokinesis [51], further support the presence of active chromosomal regulation. CCNA2 regulates S-phase progression and the G2/M transition [52], while CKAP2L is associated with spindle organization and microtubule dynamics [36]. The inclusion of TP53, a central regulator of DNA damage response and apoptosis [53], suggests integration between cell-cycle arrest, genomic stability, and differentiation-associated stress responses. In this network-based context, these genes likely reflect transitional cellular states in which proliferative programs are progressively downregulated while differentiation programs are established. Functional enrichment analysis further supports the involvement of energy-dependent and structural reorganization processes. GO terms such as ATP binding, microtubule binding, and DNA-dependent ATPase activity indicate enrichment of chromatin remodeling and cytoskeletal regulation functions [[54], [55], [56], [57], [58], [59]]. Biological process terms highlight metabolic reprogramming, including organic substance metabolism, nitrogen compound metabolism, and macromolecule biosynthesis, suggesting increased biosynthetic and energetic demand during differentiation [30,31,60,61]. Cellular component enrichment in intracellular organelles, cytoplasm, and protein complexes indicates coordinated activity across nuclear and cytoplasmic compartments, consistent with integrated regulation of transcription, translation, and protein processing during lineage commitment [37,38].

KEGG pathway analysis reinforces these observations, with significant enrichment of ribosomal pathways and central carbon metabolism, including glycolysis and oxidative phosphorylation [9,39,42]. These findings suggest increased translational demand and energy production requirements, consistent with a metabolically active differentiation state. Overall, these results support a systems-level model in which metabolic remodeling and protein synthesis are tightly linked to the progression from pluripotency toward neuronal identity [40,41]. The protein–protein interaction network reveals a modular architecture dominated by a central cluster enriched in cell-cycle regulation, DNA replication, and stress response pathways. This hub-dominated structure suggests that proliferative signaling constitutes a core regulatory backbone during early differentiation stages in the bioinformatic framework. Peripheral clusters, by contrast, are enriched in more specialized pathways, including meiotic processes and developmental signaling, indicating functional diversification across network modules. The presence of PI3K-Akt, FoxO, HIF-1, and microRNA-related signaling pathways further suggests integration of survival signaling, metabolic adaptation, and post-transcriptional regulation within the same regulatory landscape. Collectively, this modular organization is consistent with a putative hierarchical organization in which central proliferative hubs coordinate broader signaling networks while peripheral modules contribute to lineage-specific differentiation processes [43,45,[62], [63], [64], [65]].

Predicted miRNA–hub gene interactions indicate an additional layer of post-transcriptional regulation. Broadly connected miRNAs such as hsa-miR-3613-5p and hsa-miR-329-5p may function as higher-order regulators targeting multiple genes involved in both cell-cycle progression and checkpoint control, including CCNA2, CDK1, TP53, and BRCA1 [46,[66], [67], [68]]. Such coordinated targeting may suggest a potential mechanism for synchronized cell-cycle exit during neuronal differentiation. Furthermore, miRNA-mediated regulation of cytoskeletal and spindle-associated genes may imply a broader role in coordinating structural remodeling required for neuronal polarization and neurite extension. However, these interactions remain predictive and require experimental validation. Promoter motif analysis provided an exploratory view of potential cis-regulatory features associated with the prioritized hub genes. Enrichment of motifs associated with transcriptional activation, chromosome organization, and Wnt signaling suggests involvement of developmental regulatory pathways [[47], [48], [49], [50],^69^]. Cell-cycle genes such as CDK1 and AURKB exhibit motif patterns consistent with tightly regulated transcriptional control, whereas genes such as FAM83D may represent distinct regulatory nodes potentially linked to Wnt-mediated developmental signaling [[70], [71], [72]]. Although some motifs were linked to developmental and signaling-related annotations, these associations should be interpreted cautiously because motif-based functional assignments can be influenced by database coverage, motif similarity, and non-specific sequence features. Therefore, the promoter motif results are best viewed as candidate regulatory signatures that require validation by transcription factor binding data, such as ChIP-seq, ATAC-seq, or reporter assays.

Codon usage analysis further indicates moderate codon bias across hub genes, with CAI values suggesting relatively efficient translational potential and ENC values indicating non-random but balanced codon usage. Observed correlations between GC content, GC3S, and CAI suggest that nucleotide composition may contribute to codon preference patterns in this gene set [[73], [74], [75], [76]]. RSCU analysis supports preferential usage of specific synonymous codons, implying potential fine-tuning of translational efficiency. However, these findings should be interpreted cautiously as bioinformatic inferences of translational tendencies rather than direct evidence of evolutionary selection or functional optimization [77,78].

Despite providing a multi-layered systems-level view of motor neuron differentiation, this study is limited by its reliance on in silico inference. Hub gene identification is dependent on network topology and may not reflect causal regulatory importance. Protein–protein interaction data are derived from curated databases that may not fully capture context-specific interactions in differentiating neuronal systems. Functional enrichment analyses are subject to annotation bias and do not directly measure biological activity. Similarly, miRNA–gene and promoter motif predictions represent putative regulatory relationships that require experimental validation. Codon usage metrics infer translational potential but do not directly assess protein expression dynamics. Additionally, the key findings were not benchmarked against an independent iPSC-derived motor neuron differentiation dataset. Therefore, all results should be interpreted as hypothesis-generating rather than confirmatory.

Based on these findings, several testable predictions can be proposed. First, candidate hub genes such as BUB1, AURKB, CCNA2, CDK1, and TP53 should be examined across independent iPSC-to-motor neuron differentiation datasets to determine whether their expression is stage-specific, transient, or associated with residual proliferative populations. Second, perturbation of these hub genes may disrupt differentiation efficiency or neuronal maturation markers. Third, predicted miRNAs such as hsa-miR-3613-5p may modulate cell-cycle exit and differentiation efficiency. Fourth, Wnt-associated regulatory motifs may respond to pathway modulation, influencing genes such as FAM83D. Finally, codon usage bias may correlate with measurable differences in translational efficiency during differentiation. In conclusion, this study proposes a systems-level model in which motor neuron differentiation from iPSCs is governed by an interconnected regulatory network integrating cell-cycle control, metabolic reprogramming, transcriptional regulation, and post-transcriptional modulation. Rather than defining definitive biological mechanisms, the findings generate a set of coherent bioinformatic hypotheses describing how proliferative and differentiation programs may be coordinated at multiple regulatory levels. Experimental validation will be essential to determine whether these inferred interactions contribute causally to motor neuron development and whether they can be exploited to improve differentiation protocols or disease modeling systems.

## 5 Conclusion

This bioinformatics study provides an integrated analysis of transcriptomic data to explore molecular networks and regulatory signatures potentially involved in the differentiation of induced pluripotent stem cells (iPSCs) into motor neurons (MNs). Fifteen hub genes were identified, including BUB1, BUB1B, TOP2A, AURKB, CCNA2, CKAP2L, and TP53, which showed strong connectivity and were mainly enriched in cell cycle and mitotic regulation pathways, suggesting a potential association between genomic stability-related processes and neuronal differentiation.

Functional enrichment analyses indicated the involvement of translational and metabolic processes, such as ribosomal biogenesis and energy-related pathways, reflecting the high biosynthetic demand during neuronal development. In addition, regulatory analyses revealed potential transcriptional control through conserved promoter motifs and cis-regulatory elements, as well as post-transcriptional regulation via predicted miRNA–mRNA interactions. Codon usage bias analysis further suggested that nucleotide composition and codon preference patterns may influence translational tendencies during differentiation.

Overall, these results provide a computational framework for understanding potential molecular mechanisms underlying iPSC-derived MN differentiation. However, the findings remain predictive in nature and require experimental validation to confirm their biological relevance and functional roles.

## Ethics approval and consent to participate

The study involved only plant materials (*Solanum lycopersicum*) and did not include experiments on humans or animals; therefore, ethical approval was not required.

## Consent for publication

The research does not contain any person's data in any form, and all authors have consent for publication. There were no human participants, so there is no need for participants to consent to publish.

## Funding information

This research did not receive any specific grant from funding agencies in the public, commercial, or not-for-profit sectors.

## CRediT authorship contribution statement

**Maryam Sadeghi:** Methodology, Software, Writing – original draft. **Shima Hadifar:** Data curation, Validation, Writing – review & editing. **Abozar Ghorbani:** Methodology, Supervision, Writing – review & editing.

## Declaration of competing interests

The authors declare that they have no known competing financial interests or personal relationships that could have appeared to influence the work reported in this paper.

## Data Availability

No data was used for the research described in the article.
